# Transcriptomic analysis in tomato fruit reveals divergences in genes involved in cold stress response and fruit ripening

**DOI:** 10.3389/fpls.2023.1227349

**Published:** 2023-07-28

**Authors:** Oscar W. Mitalo, Seung Won Kang, Long T. Tran, Yasutaka Kubo, Tohru Ariizumi, Hiroshi Ezura

**Affiliations:** ^1^ Graduate School of Life and Environmental Sciences, University of Tsukuba, Tsukuba, Japan; ^2^ Tsukuba-Plant Innovation Research Center, University of Tsukuba, Tsukuba, Japan; ^3^ Graduate School of Environmental and Life Science, Okayama University, Okayama, Japan

**Keywords:** cold stress, chilling injury, “Micro-Tom”, “Moneymaker”, RNA-Seq, ripening

## Abstract

Cold storage is widely used to extend the postharvest life of most horticultural crops, including tomatoes, but this practice triggers cold stress and leads to the development of undesirable chilling injury (CI) symptoms. The underlying mechanisms of cold stress response and CI development in fruits remain unclear as they are often intermingled with fruit ripening changes. To gain insight into cold responses in fruits, we examined the effect of the potent ethylene signaling inhibitor 1-methylcyclopropene (1-MCP) on fruit ripening, CI occurrence and gene expression in mature green tomatoes during storage at 20°C and 5°C. 1-MCP treatments effectively inhibited ethylene production and peel color changes during storage at 20°C. Storage at 5°C also inhibited both ethylene production and peel color change; during rewarming at 20°C, 1-MCP treatments inhibited peel color change but failed to inhibit ethylene production. Furthermore, fruits stored at 5°C for 14 d developed CI symptoms (surface pitting and decay) during the rewarming period at 20°C regardless of 1-MCP treatment. Subsequent RNA-Seq analysis revealed that cold stress triggers a large-scale transcriptomic adjustment, as noticeably more genes were differentially expressed at 5°C (8,406) than at 20°C (4,814). More importantly, we have found some important divergences among genes involved in fruit ripening (up- or down-regulated at 20°C; inhibited by 1-MCP treatment) and those involved in cold stress (up- or down-regulated at 5°C; unaffected by 1-MCP treatment). Transcriptomic adjustments unique to cold stress response were associated with ribosome biogenesis, NcRNA metabolism, DNA methylation, chromatin formation/remodeling, and alternative splicing events. These data should foster further research into cold stress response mechanisms in fruits with the ultimate aim of improving tolerance to low temperature and reduction of CI symptoms during cold storage.

## 1 Introduction

Cold storage is an inevitable technique during postharvest handling of horticultural produce, mainly because of the broad spectrum inhibitory effects of low temperature on quality deterioration and postharvest loss. Low temperature is generally thought to slow most cell metabolic processes (Chinnusamy et al., 2007; Rao, 2015), including those which contribute to reduced postharvest life such as fruit ripening, respiration and senescence. Storage at low temperatures also inhibits growth of most postharvest pathogens which are popularly associated with reduced quality (Vico et al., 2010; Manning et al., 2016). However, certain crops particularly tropical and subtropical types are sensitive to low temperatures, and hence suboptimal cold storage triggers a multitude of physiological disorders collectively referred to as chilling injury (CI) (Sevillano et al., 2009). CI symptoms are complex in nature and vary depending on species, cultivar, tissue and organ, and maturity stage (Wang, 1994; Sevillano et al., 2009). As CI symptoms are deleterious and contribute greatly to horticultural crop loss and heavy economic losses, there is an ongoing effort to understand the regulatory mechanisms involved in order to develop resilient crops.

Relevant progress in understanding cold stress responses and CI development has been made in vegetative plant and organs, particularly leaves. In tolerant plants such as Arabidopsis, wheat and barley, cold stress triggers an adaptive response called cold acclimation, which refers to a suite of physiological and biochemical changes that are primarily regulated by three C-repeat binding factor/drought response element binding factor 1B (CBF/DREB1) genes (Jiang et al., 2017). Low temperature rapidly triggers the expression of *CBFs*, and the cold-induced CBF proteins activate the expression of numerous cold-regulated genes, thus enhancing tolerance to cold stress (Kidokoro et al., 2022). While cold sensitive plants generally lack the capacity to cold acclimate, a limited but relevant ability to adapt to chilling stress was reported in three-week old tomato seedlings (Barrero-Gil et al., 2016), as well as in the seedlings of maize (Anderson et al., 1994), rice (Kuk et al., 2003), and sweet pepper (Liu et al., 2001).

Unlike in the vegetative stages, cold stress responses and the mechanisms regulating CI development in the fruit remain unclear. This is partly because the adjustments that occur in response to cold stress at the vegetative stage are not necessarily similar to those taking place in the fruits of the same plant. For instance, cold stress activated the expression of *SlCBF1* in the leaves of “Micro-Tom” tomato plants but not in the fruits (Weiss and Egea-Cortines, 2009), while citrus *COR15*–which encodes a dehydrin–was swiftly induced by cold stress in leaves but expressed constitutively in fruits (Sanchez-Ballesta et al., 2004). Secondly, cold stress responses in fruits are often intermingled with fruit ripening and senescence (Biswas et al., 2016; Shipman et al., 2021), and hence it is hard to ascribe the resultant physio-molecular adjustments and symptoms to CI.

Tomato is an important horticultural crop and an established model for the study of fleshy fruit species (Ezura, 2009; Menda et al., 2013). Tomato fruits are very sensitive to cold stress and storage at temperatures below 10°C triggers several CI symptoms such as flavor loss, impaired ripening, surface pitting, poor texture, increased susceptibility to postharvest decay and discoloration (Biswas et al., 2016). As a typical climacteric fruit, the ripening process in tomato is largely controlled by the phytohormone ethylene (Li et al., 2019), through up- or down-regulation of various ripening-associated genes. Mutations in ethylene receptors have been shown to significantly inhibit fruit ripening in tomatoes (Lanahan et al., 1994; Okabe et al., 2011), but partial retention of ethylene sensitivity in these mutants likely due to functional redundancy among several receptors was also reported (Tieman et al., 2000; Gratão et al., 2012; Chen et al., 2019). 1-Methylclopropene (1-MCP) is a synthetic compound that irreversibly binds to ethylene receptors (Serek et al., 2006; Watkins, 2006), at a higher affinity than ethylene (Blankenship and Dole, 2003), thus preventing the hormone from binding to the receptors and blocking ethylene activation of downstream events. A major advantage of using 1-MCP to block ethylene signaling over other approaches is that 1-MCP indiscriminately binds to all ethylene receptors in addition to providing precision and high efficiency (Schotsmans et al., 2009; Kamiyoshihara et al., 2012), which allows researchers to study both ethylene-dependent and -independent processes in a relatively short period of time.

Here, RNA-Seq analysis was used to identify transcriptional adjustments which occur during normal ripening at 20°C or in response to cold stress (5°C, 14 d) in “Micro-Tom” tomatoes. In an attempt to differentiate cold stress-induced *versus* ethylene-induced (to a large extent, fruit ripening) changes, we also monitored gene expression changes in both “Micro-Tom” and “Moneymaker” tomatoes that had been repeatedly treated with 1-MCP during storage at 20°C and 5°C. This analysis revealed that genes associated with epigenetic modifications, ribosome biogenesis, proteasome and non-coding RNA metabolism, alternative splicing events and several transcription factors could be involved in the series of events unique to cold stress and CI development in fruits.

## 2 Materials and methods

### 2.1 Plant material and treatments

*Solanum lycopersicum* “Micro-Tom” (TOMJPF00001) and “Moneymaker” cultivars were obtained from the National Bioresource Project (MEXT, Japan) through the TOMATOMA database (Saito et al., 2011). Fruits were harvested at the mature green stage (before onset of autocatalytic ethylene production), washed in commercial bleach (1:10 dilution of sodium hypochlorite) and sorted to ensure uniform size, color and absence of defects or damage. This timing of harvest was well thought out to avoid the effect of large amounts of ethylene, which are produced at later maturity stages, during cold stress tests. For each cultivar, two groups of 50 fruits were used to characterize the ripening behavior at 20°C; the first group were treated (2–3 times a week) with 2 µLL^-1^ 1-MCP for 12 h, while the second group were a non-treated control. For cold stress tests, two groups of 50 fruits were also used; the first group was treated with 1-MCP as described above while the other group was a non-treated control. “Micro-Tom” fruits were stored at 5°C for 14 d before being transferred to 20°C for up to 21 d. Three separate storage trials were carried out on “Micro-Tom” fruits. “Moneymaker” fruits were also stored at 5°C but for 21 d; after every 7 d, 10 fruits were transferred to 20°C to observe CI symptoms. 1-MCP treatments were carried out to keep the fruits insensitive to ethylene. To release 1-MCP gas, SmartFresh™ powder (AgroFresh, PA, USA) was dissolved in water and soda lime was added in the sealed treatment containers to reduce CO_2_ accumulation. For DNA methylation inhibitor treatment, “Moneymaker” fruits at the mature green stage were used. The fruits were injected with 100 µL of 50 mM 5-azacytidine aqueous solution into the columella at harvest and every 7 d during storage at 5°C. The negative controls were injected with the same amount of distilled water. In all treatments, pericarp samples of three replicate fruits were collected, frozen in liquid nitrogen and stored at -80°C for future analysis.

### 2.2 Determination of peel color

Peel color measurements were carried out on four evenly distributed equatorial sites using a Konica Minolta Color Reader CR-10 (Konica Minolta, Tokyo, Japan). The Hunter lab parameter a*, which is a measure of greenness or redness, was recorded and then expressed as the mean of six replicate fruits.

### 2.3 Ethylene measurements

Individual fruits were incubated at the respective storage temperatures for 1 h. Headspace gas (1 mL) was then withdrawn and injected into a Shimadzu 5890 series gas chromatograph (Shimadzu, Kyoto, Japan) equipped with a flame ionization detector (200°C) and an activated alumina column (80°C). Ethylene production rates were expressed as the mean of 10 replicate fruits.

### 2.4 Determination of CI index

Fruits were assessed visually for severity of CI symptoms based on a five-point scale (0 = no injury; 1 = < 10%; 2 = 11 to 25%; 3 = 26–40%, and 4 = > 40%) consisting of three parameters: surface pitting, uneven ripening, and decay (Vega-García et al., 2010; Albornoz et al., 2019). The CI index was then calculated by determining the average of the injury levels of surface pitting, uneven ripening, and decay. For each time point, 10 fruits were evaluated individually, and the CI indexes were averaged.

### 2.5 Library construction and RNA sequencing

Pericarp samples of “Micro-Tom” fruits collected at harvest (0 d), after 14 d storage at either 5°C or 20°C, and after 21 d at 20°C or 14 d at 5°C followed by 7 d at 20°C (three fruit per treatment) were used for RNA-Seq analysis. Total RNA was extracted from 100 mg samples using the RNeasy^®^ Plant Mini Kit (Qiagen, Hilden, Germany), treated with DNase I (Nippon Gene, Tokyo, Japan) to remove genomic DNA contamination and further purified with FavorPrep after Tri-Reagent RNA Clean-up Kit (Favorgen Biotech. Co., Ping-Tung, Taiwan). Paired-end libraries were then constructed using NEBNext^®^ Ultra^™^ II Directional RNA Library Prep Kit (New England Biolabs), and sequencing was performed on an Illumina Novaseq 6000 platform (Illumina, Inc.).

### 2.6 Differential gene expression analysis

The sequenced reads were analyzed primarily on the Galaxy online platform^1^. Trimming was first carried out to exclude both low quality sequences and adapter sequences. The trimmed reads were then mapped to the reference *S. lycopersicum* genome (SL4.0), and mapped reads were counted using the featureCounts tool. Gene expression levels were then normalized as transcripts per kilobase million (TPM) reads. Differentially expressed genes (DEGs) were obtained by comparing the expression levels in samples after 14 d of storage at either 5°C or 20°C with those of at-harvest (0 d) samples on the iDEP (v. 0.96) web-based toolkit (Ge et al., 2018). Three criteria were used to detect DEGs: (i) TPM ≥ 1.0 in either of the three replicate samples, (ii) false discovery rate ≤ 0.01, and (iii) two-fold increase or increase in expression levels. Weighted gene co-expression network analysis (WGCNA) method (Langfelder and Horvath, 2008) was then employed to generate clusters of highly correlated genes with 8 and 0.15 as thresholding power and tree-cut parameters, respectively. Significantly enriched gene ontology (GO) terms and KEGG pathways were established using the ShinyGO (v. 0.77) web-based toolkit (Ge et al., 2020). The cut-off for significantly enriched terms was *P* < 0.05.

### 2.7 Quantitative real-time PCR (qPCR) analysis

cDNA was synthesized from 1 µg of clean DNase I-treated RNA (same samples as those used for library construction) using the SuperScript^®^ III First-Strand Synthesis SuperMix for qRT-PCR kit (Invitrogen). Gene-specific primers (**Supplementary Table 1**) were designed using the Primer3 online software (version 0.4.0^2^). Gene expression of three biological replicates was examined on a Stratagene Mx3005P Real-Time QPCR System (Agilent Technologies, Santa Clara, CA, USA) using KOD SYBR^®^ qPCR Mix (Toyobo, Osaka, Japan). *SlActin* (*Solyc03g078400*) was used as the housekeeping gene after examining its constitutive expression pattern from the RNA-Seq data. Relative expression values were calculated using the 2^-ΔΔCt^ method with at-harvest (0 d) samples calibrated as 1.

### 2.8 McrBC-qPCR analysis

Genomic DNA was extracted from the pericarp of “Moneymaker” fruits (three replicates for each time point) using the Nucleospin^®^ Plant II kit (Takara, Shiga, Japan).The genomic DNA (1 µg) was then digested at 37°C overnight with McrBC (Takara), before performing qPCR analysis as described in section 2.7 with 20 ng digested DNA as a template. Undigested gDNA samples were used as controls. Relative methylation was then calculated as 2^Ct(digested) - Ct(undigested)^, such that higher relative McrBC-qPCR signals correspond to higher methylation levels.

### 2.9 Gene expression analysis in tomato leaves

Three-week old “Moneymaker” seedlings were transferred to 5°C or 20°C for up to 7 d. Leaves were collected at 0, 3, 5, and 7 d for RNA extraction and qPCR analysis as described in section 2.7.

### 2.10 Statistical analysis

Data obtained in this study were subjected to statistical analysis using R v.3.4.0^3^. Differences in ethylene production rates, peel color, CI index, methylation levels and gene expression levels were determined using ANOVA followed by Tukey’s *post hoc* tests.

## 3 Results

### 3.1 Characterization of fruit ripening, chilling injury and the effect of 1-MCP

**Figure 1:**
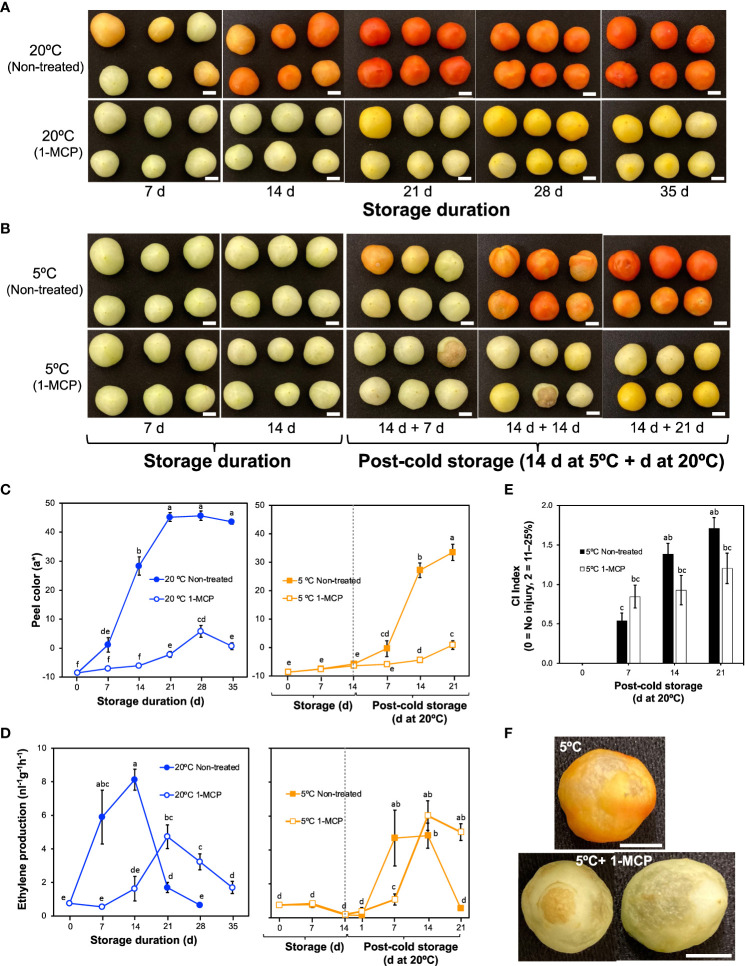
External changes and ethylene production rates in mature green “Micro-Tom” tomato fruits during storage at 20 and 5°C with or without 1-methylcyclopropene (1-MCP) treatment. **(A)** Effect of 1-MCP on external appearance during storage at 20°C. **(B)** Effect of 1-MCP on external changes during storage at 5°C and post-cold storage at 20°C. **(C)** Changes in CIELAB color parameter a* during storage at 20°C (left panel) and 5°C (right panel) with or without 1-MCP treatment. Each datapoint represents the average (± SE) of 6 fruits. **(D)** Ethylene production rates. Each datapoint represents the average (± SE) of 10 fruits. **(E)** Chilling injury index in fruits during storage at 20°C following 14 d at 5°C. Each column represents the average (± SE) of 10 fruits. **(F)** Images of fruits showing uneven ripening and surface pitting after storage at 5°C, and decay with surface pitting in 1-MCP treated fruits. Fruits were stored at 5°C for 14 d followed by post-cold shelf life at 20°C for 7 d. Different letters in **(C, D, E)** indicate significant differences in ANOVA (Tukey’s test, *P* < 0.05). White horizontal bars in **(A, B, F)** indicate 1 cm.

Non-treated tomato fruits started to lose their green peel color after 7 d at 20°C, attaining a uniform red color after 21 d (**Figure 1A**; **Supplementary Figure 1**). This was evidenced by an increase in parameter a* from negative values to positive values (**Figure 1C**; **Supplementary Figure 1**). The changes in peel color were consistent with ethylene production rates (**Figure 1D**), which increased from 7 d in fruits during storage at 20°C and peaked at 14 d in a typical climacteric pattern. For the 1-MCP-treated fruits, both peel color changes and ethylene production rates did not change significantly in the first 14 d at 20°C; although ethylene production rates increased later on, the peak was 58% lower than that of non-treated fruits, and the fruits never attained a red color throughout the 35 d storage period.

During storage at 5°C, both non-treated and 1-MCP-treated fruits did not show any noticeable change in peel color (**Figures 1B, C**), and the ethylene production rates also did not change significantly (**Figure 1D**). On rewarming at 20°C, ethylene production rates increased, with a lag of about 6 d in 1-MCP-treated fruits (**Figure 1D**). The rewarmed fruits, however, failed to ripen normally, developed pitted surfaces and showed decay symptoms as evidenced by the high CI index values (**Figures 1B, E, F**; **Supplementary Figure 1**).

### 3.2 Transcriptomic responses in the pericarp: cold response *versus* fruit ripening

**Figure 2:**
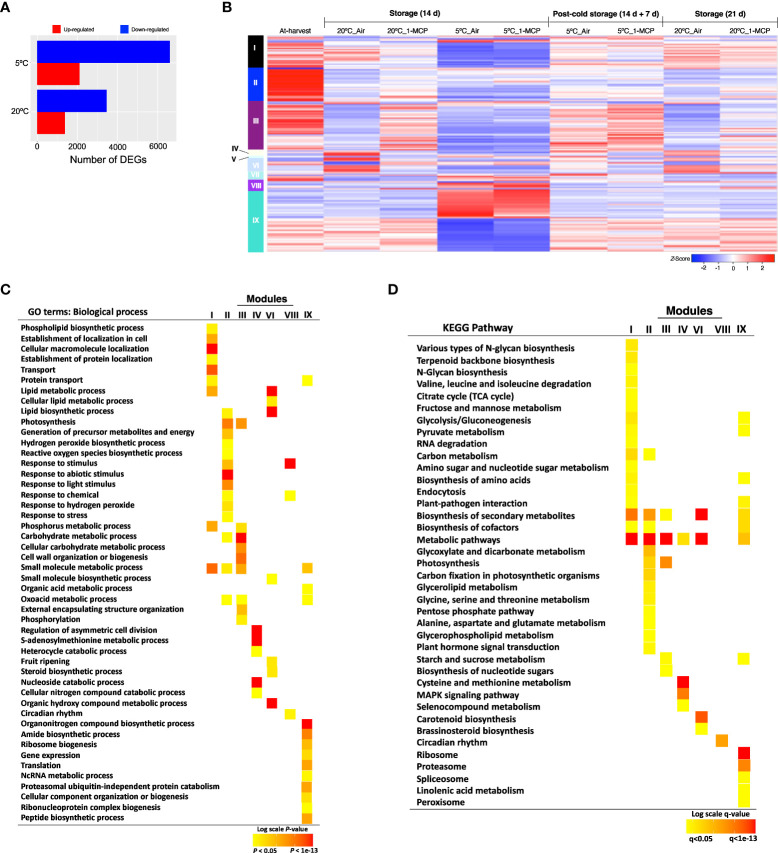
Gene expression changes in mature green “Micro-Tom” tomato fruits during storage at 20 and 5°C, and the effect of 1-MCP treatment. **(A)** Numbers of up- and down-regulated genes during storage at 20 and 5°C. Samples after 14 d of storage were compared to those collected at-harvest (0 d). **(B)** Heatmap visualization of the highly co-expressed gene clusters. **(C)** GO terms enriched among the differentially expressed genes in each cluster. Color panels indicate the *P*-value of GO enrichment. **(D)** KEGG pathway enrichment analysis among the differentially expressed genes in each cluster. Color panels indicate the significance level of enrichment.

RNA-Seq analysis was then performed to identify gene expression changes during normal fruit ripening (at 20°C) and in response to cold stress (triggered by storage at 5°C for 14 d) in “Micro-Tom” fruits. By comparing samples collected after 14 d storage at 20°C and 5°C with at-harvest (0 d) samples, we identified 10,042 DEGs. Interestingly, we found more upregulated and downregulated genes at 5°C (8,406) than at 20°C (4,814) (**Figure 2A**). Subsequent hierarchical clustering of the DEGs against temporal expression patterns in at-harvest, stored and rewarmed samples outlined 9 major modules (**Figure 2B**; **Supplementary Tables 2**-**10**). Among them, modules I, II, III, IV, VII and VIII comprised genes which were differentially expressed both at 20°C and 5°C, and 1-MCP treatment affected their expression levels at both temperatures; this indicated that they are regulated by either ethylene or low temperature. Genes in modules V and VI were up- or down-regulated during storage at 20°C, a change that was reversed by 1-MCP treatment; these expression changes were suppressed during storage at 5°C, but they recovered (at least to some extent) upon rewarming. Interestingly, module IX genes displayed insignificant expression changes during storage at 20°C, but they were up- or down-regulated at 5°C in both 1-MCP-treated and non-treated samples with a reversion to original levels upon rewarming at 20°C.

To further understand the molecular changes that are induced during cold response *versus* fruit ripening, we performed GO term and KEGG pathway-based enrichment analyses of the DEGs in each of the modules identified above. GO analysis confirmed that genes in module VI were associated with ‘fruit ripening’ (**Figure 2C**), especially the ‘carotenoid biosynthesis pathway’ (**Figure 2D**). On the other hand, module IX genes were dominated by functions such as ‘gene expression’, ‘translation’, ‘ribosome biogenesis’, ‘proteasome’ and ‘NcRNA metabolism’ (**Figures 2C, D**). Other functions that were uniquely enriched in module IX genes included ‘spliceosome’, ‘organonitrogen biosynthesis’, and ‘linolenic acid metabolism’.

All in all, our results indicated that there was a divergence in the molecular adjustments triggered by cold response and those involved in fruit ripening in tomatoes. Module VI genes, in particular, were mostly involved in changes that lead to fruit ripening and they were under the influence of ethylene signaling (i.e., affected by 1-MCP treatment). Conversely, module IX genes were regulated by low temperature while ethylene signaling had little or no significant influence on their expression levels (i.e., unchanged by ethylene at 20°C and unaffected by 1-MCP at 5°C.

### 3.3 Transcripts associated with fruit ripening

**Figure 3:**
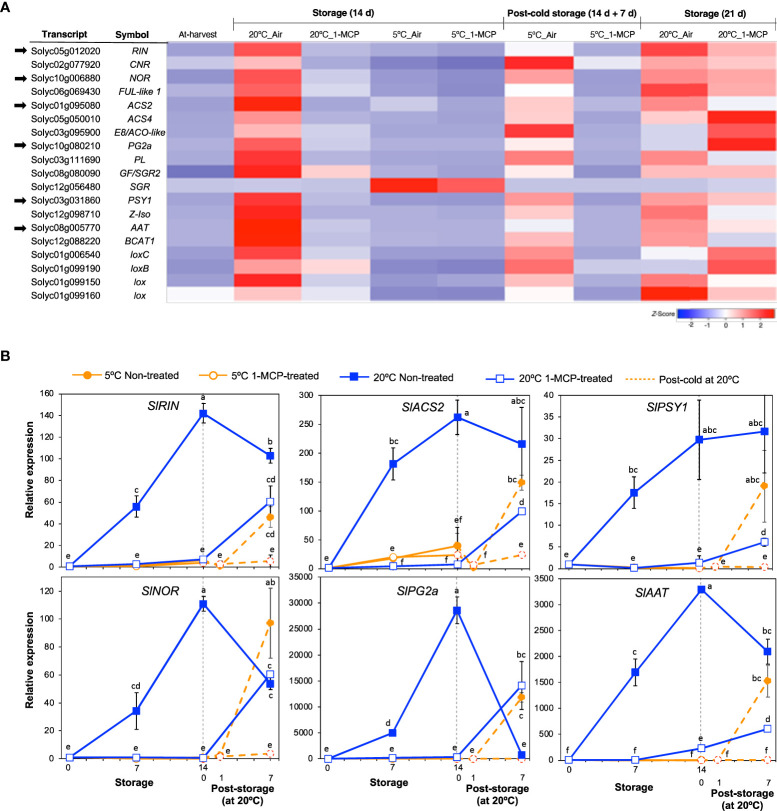
Expression patterns of selected genes commonly associated with fruit ripening and the effect of 1-MCP. **(A)**. Heatmap of DEGs encoding transcription factors and enzymes in mature green “Micro-Tom” tomato fruits at the indicated storage temperatures and duration. **(B)** RT-qPCR analysis of selected genes from **(A)** in mature green “Micro-Tom” tomato fruits at the indicated times. Expression values are relative to the value at harvest (0 d) and the housekeeping gene was *SlActin*. Datapoints indicate means (± SE) of three replicate fruits. Different letters indicate significant differences in ANOVA (Tukey’s test, *P* < 0.05).

Genes that have been commonly associated with fruit ripening were selected from among the DEGs for further examination and to assert the inhibitory effect of 1-MCP. Most of these genes belonged to module VI; they were upregulated at 20°C in accordance with ethylene production rates and 1-MCP treatment inhibited this change (**Figure 3A**). However, one gene (*SlSGR*, *Solyc12g056480*), which encodes magnesium dechelatase that is involved in chlorophyll degradation (Shimoda et al., 2016), was upregulated only at 5°C and 1-MCP treatment did not inhibit this expression change, indicating that it responded only to low temperature and was independent of ethylene signaling.

Six of the genes in **Figure 3A** were then selected for validation by qPCR analysis. These included *ripening inhibitor* (*SlRIN*) and *non-ripening* (*SlNOR*) which encode established molecular regulators of fruit ripening, *1-aminocyclopropane-1-carboxylic acid synthase 2* (*SlACS2*) which encodes an ethylene biosynthetic enzyme, *polygalacturonase 2a* (*SlPG2a*) which is involved in fruit softening, *phytoene synthase 1* (*SlPSY1*) which is involved in carotenoid metabolism and the aroma volatile production-associated *alcohol acyl transferase* (*SlAAT*). As expected, the expression levels of these six genes increased markedly in non-treated fruits during storage at 20°C (**Figure 3B**), which corresponded with the ethylene production patterns observed earlier (**Figure 1D**). In 1-MCP-treated fruits at 20°C, little or insignificant changes in expression levels were registered during the first 14 d; increases however occurred at 21 d as ethylene production levels rose. The expression levels of these ripening-associated genes (except for *SlSGR*) remained mostly unchanged during storage at 5°C and even after 1 d of rewarming at 20°C; significant transcript accumulation occurred after post-cold storage for 7 d at 20°C. Together, these results reaffirmed that ethylene signaling regulates the ripening process in tomatoes and 1-MCP treatment effectively blocks ethylene-mediated regulation of the genes involved.

### 3.4 Transcripts associated with oxidative damage

**Figure 4:**
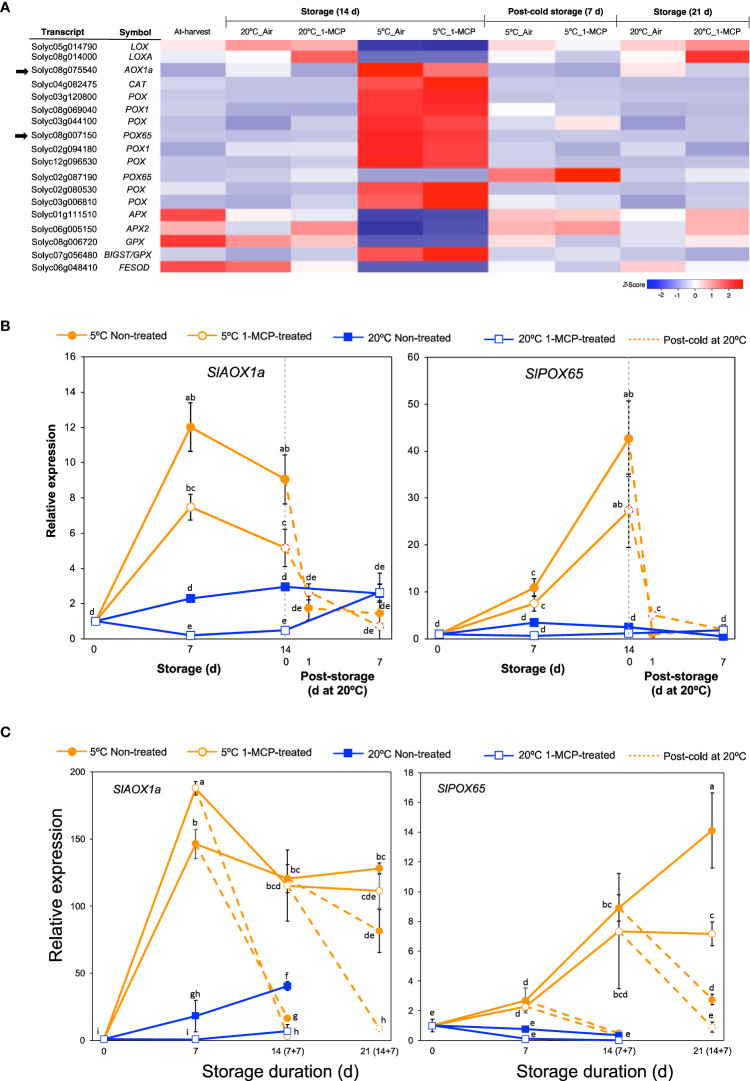
Expression patterns of genes associated with oxidative damage. **(A)** Heatmap of DEGs responding to low temperature alone in mature green “Micro-Tom” tomato fruits at the indicated storage temperatures and duration. **(B)** RT-qPCR analysis of selected genes from **(A)** in mature green “Micro-Tom” tomato fruits at the indicated times. **(C)** RT-qPCR analysis of selected genes from **(A)** in mature green “Moneymaker” tomato fruits at the indicated times. Values in brackets on the horizontal axis indicate number of days at 5°C + 7 days of rewarming at 20°C. Expression values are relative to the value at harvest (0 d) and the housekeeping gene was *SlActin*. Datapoints in **(B, C)** indicate means (± SE) of three replicate fruits. Different letters indicate significant differences in ANOVA (Tukey’s test, *P* < 0.05).

Cold stress results in increased levels of reactive oxygen species which cause oxidative damage in fruits and vegetables (Sevillano et al., 2009; Valenzuela et al., 2017). To avoid or tolerate this oxidative damage, several enzymatic reactions catalyzed by lipoxygenases (LOX), peroxidases (POX), alternative oxidases (AOX), catalases (CAT), and superoxide dismutases (SOD) are generated in the affected plants. We therefore examined further the expression patterns of genes encoding these antioxidant defense enzymes from among the DEGs. Interestingly, out of the 18 genes that we found (**Figure 4A**), a majority (13 genes, 72%) belonged to module IX as they showed insignificant changes in expression levels during storage at 20°C, but they were differentially expressed at 5°C with or without 1-MCP treatment. Furthermore, only 2 out of these 13 genes were downregulated while the rest (11 genes) were upregulated in fruits stored at 5°C.

We then studied the expression of *SlAOX1a* (*Solyc08g075540*) and *SlPOX65* (*Solyc08g007150*) in both “Micro-Tom” and “Moneymaker” fruits during storage at 5°C and 20°C via qPCR analysis. As indicated in **Supplementary Figure 1**, “Moneymaker” fruits also exhibited CI symptoms during rewarming at 20°C following storage at 5°C for 7, 14, and 21 d. *SlAOX1a* and *SlPOX65* transcript levels notably increased in both “Micro-Tom” and “Moneymaker” fruits during storage at 5°C with or without 1-MCP treatment, and decreased immediately after the fruits were rewarmed to 20°C (**Figures 4B, C**). During storage at 20°C, the expression levels remained mostly unchanged except for a relatively slight but relevant increase in *SlAOX1a* transcript levels in “Moneymaker” fruits. Therefore, it appears that the adaptive responses to cold stress-triggered ROS accumulation in tomato fruits are typically not influenced by ethylene signaling.

### 3.5 Transcripts associated with ribosome biogenesis

**Figure 5:**
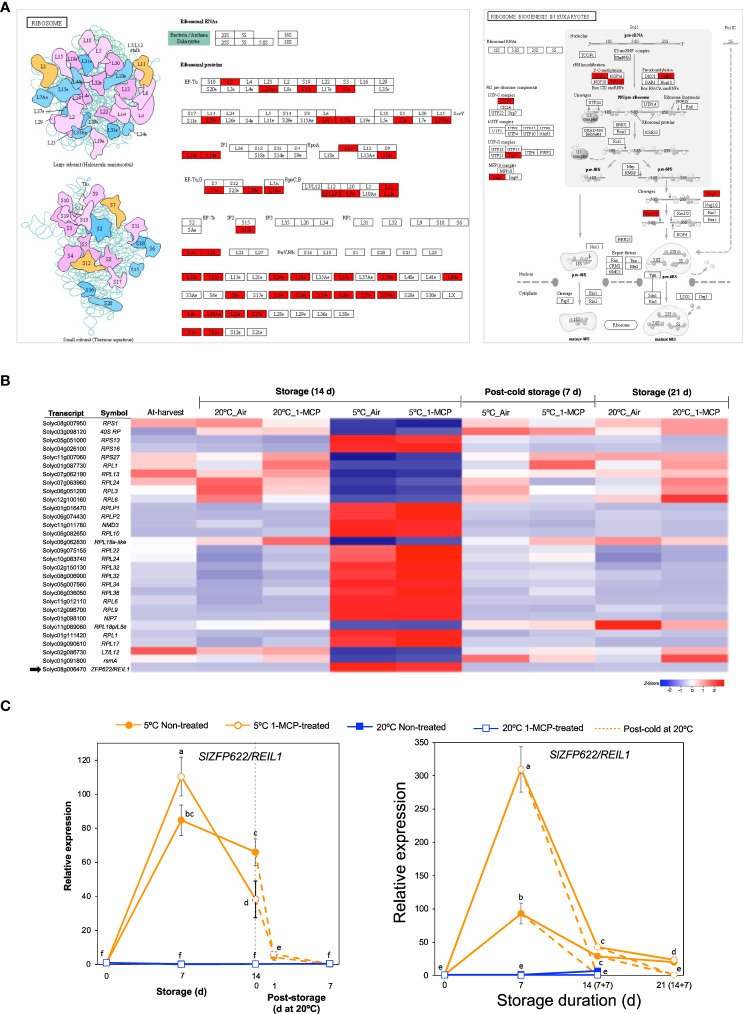
Transcriptional changes associated with ribosome biogenesis. **(A)**. Enrichment of pathways related with ribosomal proteins and their biogenesis. **(B)** Heatmap of DEGs responding to low temperature alone in mature green “Micro-Tom” tomato fruits at the indicated storage temperatures and duration. **(C)** RT-qPCR analysis of *ZFP622/REIL2* (indicated by a black arrow in **B**) in mature green “Micro-Tom” tomato fruits (left panel) and mature green “Moneymaker” tomato fruits (right panel) at the indicated times. Expression values are relative to the value at harvest (0 d) and the housekeeping gene was *SlActin*. Values in brackets on the horizontal axis indicate number of days at 5°C + 7 days of rewarming at 20°C. Datapoints indicate means (± SE) of three replicate fruits. Different letters indicate significant differences in ANOVA (Tukey’s test, *P* < 0.05).

Term enrichment analysis of our RNA-Seq data (**Figures 2C, D**) indicated that one of the molecular responses to low temperature alone (with respect to fruit ripening) was adjustments among ribosomal proteins and the ribosome biogenesis pathway in addition to ‘NcRNA metabolism’. Visualization of enriched pathways suggested that low temperature triggers comprehensive changes in both large and small ribosomal subunits as well as ribosomal biogenesis factors (**Figure 5A**). After a closer look at the DEGs, we found 30 genes belonging to module IX (**Figure 5B**). Their expression levels did not change significantly during storage at 20°C, but 18 of them were upregulated while the remaining 12 were downregulated during storage at 5°C both in 1-MCP-treated and non-treated fruits. qPCR analysis further confirmed the upregulation of *SlZFP622/REIL1* (*Solyc08g006470*), encoding a ribosome biogenesis factor, during storage at 5°C with transcript levels peaking at 7 d in both “Micro-Tom” and “Moneymaker” tomatoes (**Figure 5C**).

### 3.6 Transcripts associated with the spliceosome

**Figure 6:**
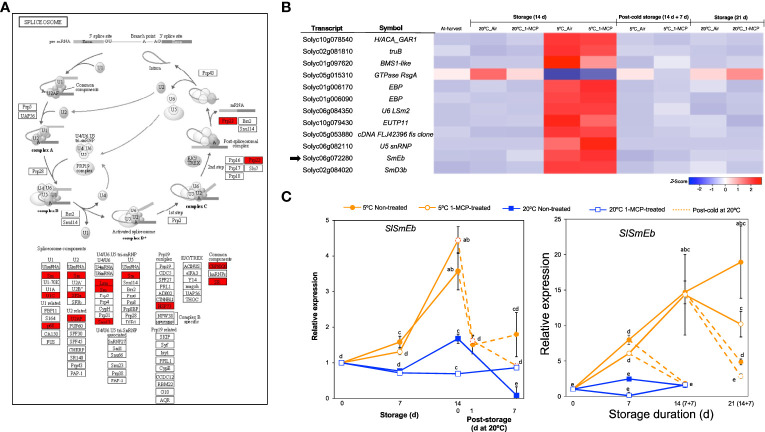
Transcriptional changes associated with the spliceosome. **(A)**. Enrichment of spliceosome-associated components among DEGs responding to low temperature alone. **(B)** Heatmap of DEGs associated with the spliceosome in mature green “Micro-Tom” tomato fruits at the indicated storage temperatures and duration. **(C)** RT-qPCR analysis of the spliceosome component *SlSmEb* (indicated by black arrow in **B**) in mature green “Micro-Tom” tomato fruits (left panel) and mature green “Moneymaker” tomato fruits (right panel) at the indicated times. Expression values are relative to the value at harvest (0 d) and the housekeeping gene was *SlActin*. Values in brackets on the horizontal axis indicate number of days at 5°C + 7 days of rewarming at 20°C. Datapoints indicate means (± SE) of three replicate fruits. Different letters indicate significant differences in ANOVA (Tukey’s test, *P* < 0.05).

Another category that stood out among the DEGs responding to low temperature alone (module IX) was ‘spliceosome’ (**Figure 2D**), a protein complex in which introns are removed from immature mRNAs to generate uninterrupted open reading frames for translation or to produce different splicing variants of the same gene and hence potentially increase the total number of proteins in the cell (Reddy et al., 2013; Wang et al., 2022). Further visualization of the spliceosome pathway confirmed that several components were highly enriched among the module IX DEGs (**Figure 6A**). Twelve genes associated with the spliceosome were then isolated from the total DEGs (**Figure 6B**), of which 11 were upregulated only in fruits stored at 5°C for 14 d with or without 1-MCP treatment while 1 gene was downregulated. This included *SlSmEb* (*Solyc06g072280*) whose ortholog in Arabidopsis has been associated with proper alternative splicing events during chilling stress responses (Wang et al., 2022). By qPCR analysis, we confirmed that *SlSmEb* transcripts accumulate in both “Micro-Tom” and “Moneymaker” tomatoes during storage at 5°C irrespective of 1-MCP treatments, but little or insignificant changes in expression occur at 20°C (**Figure 6C**). The cold-specific upregulation of *SlSmEb* is further supported by the swift drop in expression levels (within 1 d in “Micro-Tom” tomatoes) when fruits were transferred from 5°C to 20°C.

### 3.7 Transcripts associated with transcription factors, chaperones and signaling peptides

**Figure 7:**
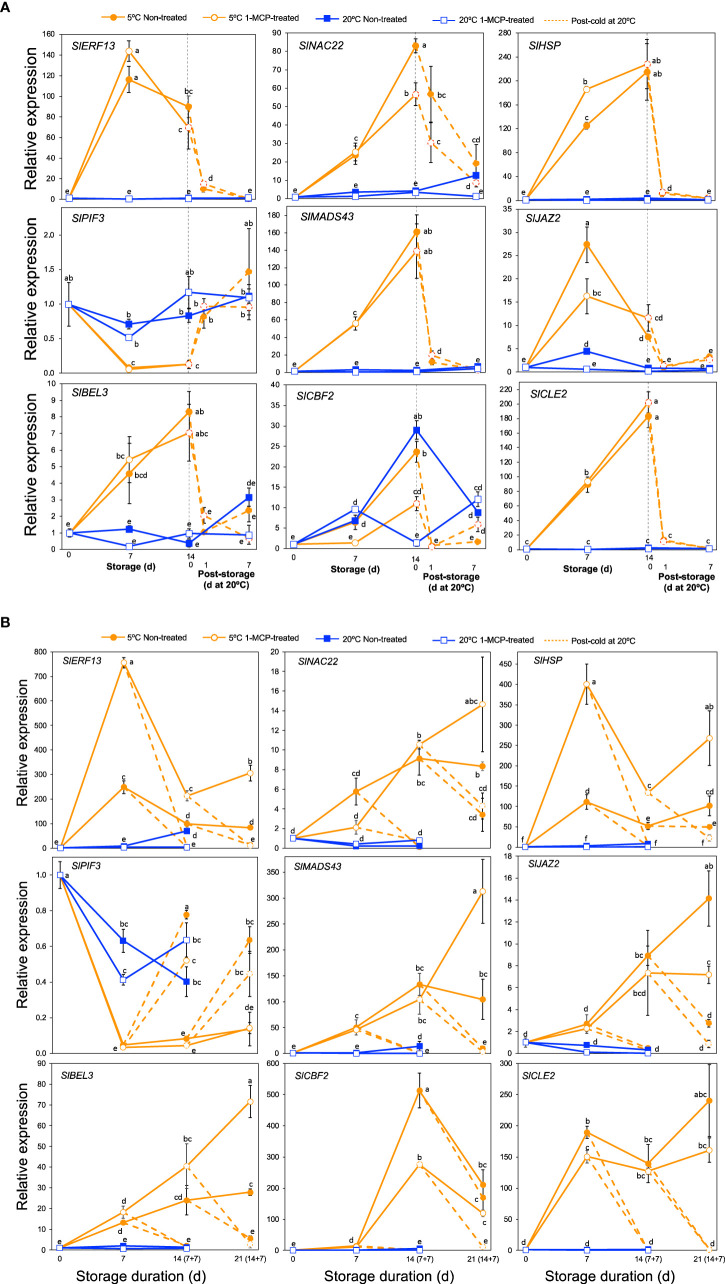
Expression patterns of genes encoding various transcription factors, chaperones and signaling peptides. **(A)**. RT-qPCR analysis in mature green “Micro-Tom” tomato fruits. **(B)** RT-qPCR analysis in mature green “Moneymaker” tomato fruits. Expression values are relative to the value at harvest (0 d) and the housekeeping gene was *SlActin*. Values in brackets on the horizontal axis in B indicate number of days at 5°C + 7 days of rewarming at 20°C. Datapoints indicate means (± SE) of three replicate fruits. Different letters indicate significant differences in ANOVA (Tukey’s test, *P* < 0.05) *SlERF13* (*Solyc10g076370*), *SlNAC22* (*Solyc09g025310*), *SlHSP* (*Solyc06g054150*), *SlPIF3* (*Solyc01g102300*), *SlMADS43* (*Solyc05g013370*), *SlJAZ2* (*Solyc12g009220*), *SlBEL3* (*Solyc04g080790*), *SlCBF2* (*Solyc03g124110*) and *SlCLE2* (*Solyc01g098890*).

As is the case with other biotic and abiotic stresses, the physiological and biochemical alterations which occur in plants under cold stress are controlled by several regulatory hubs that include various transcription factors, hormones, chaperones and signaling peptides (Kosová et al., 2018; Mehrotra et al., 2020). We therefore scrutinized our DEGs particularly in module IX to see how genes associated with these regulatory hubs are affected by cold stress. This led to the identification of 99 DEGs of which 60 encoded various transcription factor families, 14 were associated with jasmonate and auxin signaling, 10 encoded heat shock proteins, 13 were related with calcium signaling and 2 encoded clavata3/embryo surrounding region-related (CLE) signaling peptides (**Supplementary Table 11**). qPCR analyses were then performed on a selected nine genes to validate the RNA-Seq data and to evaluate their expression patterns in response to cold stress in “Micro-Tom” and “Moneymaker” tomatoes. In both cultivars, transcripts of *SlERF13*, *SlNAC22*, *SlHSP*, *SlMADS43*, *SlJAZ2*, *SlBEL3* and *SlCLE2* increased dramatically, while those of *SlPIF3* decreased during storage at 5°C regardless of 1-MCP treatment; changes at 20°C were insignificant (**Figure 7**). Transcript levels in fruits at 5°C also dropped following transfer to 20°C. However, while *SlCBF2* transcripts increased during storage at both 5 and 20°C in “Micro-Tom” fruits (**Figure 7A**), increased expression levels were registered only at 5°C in “Moneymaker” fruits especially after 14 d (**Figure 7B**).

### 3.8 Transcripts associated with epigenetic modifications

**Figure 8:**
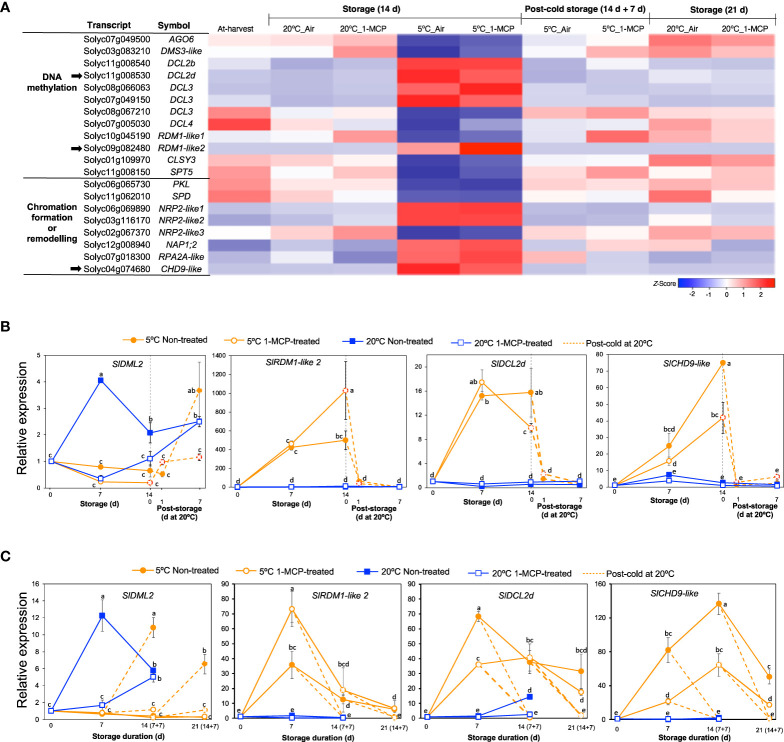
Expression patterns of genes associated with epigenetic modifications. **(A)** Heatmap of DEGs associated with DNA methylation and chromatin formation/remodelling in mature green “Micro-Tom” tomato fruits at the indicated storage temperatures and duration. **(B)** RT-qPCR analysis of selected genes (indicated by black arrow in **A**) in mature green “Micro-Tom” tomato fruits. **(C)** RT-qPCR analysis in mature green “Moneymaker” tomato fruits. Expression values are relative to the value at harvest (0 d) and the housekeeping gene was *SlActin*. Values in brackets on the horizontal axis in **(C)** indicate number of days at 5°C + 7 days of rewarming at 20°C. Datapoints indicate means (± SE) of three replicate fruits. Different letters indicate significant differences in ANOVA (Tukey’s test, *P* < 0.05).

The finding that ‘NcRNA metabolism’ was one of the highly enriched GO terms among module IX DEGs (**Figure 2C**) prompted us to also look into the expression patterns of genes associated with epigenetic modifications. Out of the 20 genes that we identified, 12 were associated with DNA methylation while 8 were associated with chromatin formation/remodeling (**Figure 8A**). Among the DNA methylation-related genes, 6 encoded short interfering RNA-producing dicer-like enzymes of which 4 were upregulated while 2 were downregulated only during storage at 5°C for 14 d regardless of 1-MCP treatments. Interestingly, we also found 2 genes that encode RNA-directed DNA methylation 1 (RDM1) protein; *SlRDM1-like 1* (*Solyc10g045190*) was slightly downregulated while *SlRDM1-like 2* (*Solyc09g082480*) was highly upregulated in both 1-MCP-treated and non-treated fruits after 14 d at 5°C. For the genes associated with chromatin formation/remodeling, 5 were upregulated while only 3 were downregulated in both 1-MCP-treated and non-treated fruits after 14 d at 5°C. All of these genes (both DNA methylation- and chromatin formation/remodeling-associated) showed insignificant expression changes during storage at 20°C and the expression changes induced at 5°C were reversed when fruit were rewarmed to 20°C.

We then selected three genes which showed high and clear expression changes based on their TPM values, that is *SlRDM1-like 2*, *dicer-like 2d* (*SlDCL2d*, *Solyc11g008530*) and *chromodomain helicase 9 (CHD9)-like* (*SlCHD9-like*, *Solyc04g074680*), for further analysis by qPCR. In addition, we also examined the expression of *Demeter-like 2* (*SlDML2*, *Solyc10g083630*) which is associated with active DNA demethylation in tomatoes (Lang et al., 2017). In both “Micro-Tom” and “Moneymaker” tomatoes, *SlDML2* transcripts increased significantly during storage at 20°C and peaked after 7 d, but this increase was inhibited in 1-MCP-treated fruits (**Figures 8B, C**). However, the expression of *SlDML2* did not change significantly during storage at 5°C, but increases were observed only after rewarming at 20°C. Transcripts of *SlRDM1-like 2*, *SlDCL2d*, and *SlCHD9-like* showed little or no significant changes during storage at 20°C, but they accumulated highly during storage at 5°C irrespective of 1-MCP treatments (**Figures 8B, C**). The increased expression registered at 5°C was reversed within 1 d (in “Micro-Tom” fruits) and after 7 d (in “Moneymaker” fruits) of rewarming at 20°C.

**Figure 9:**
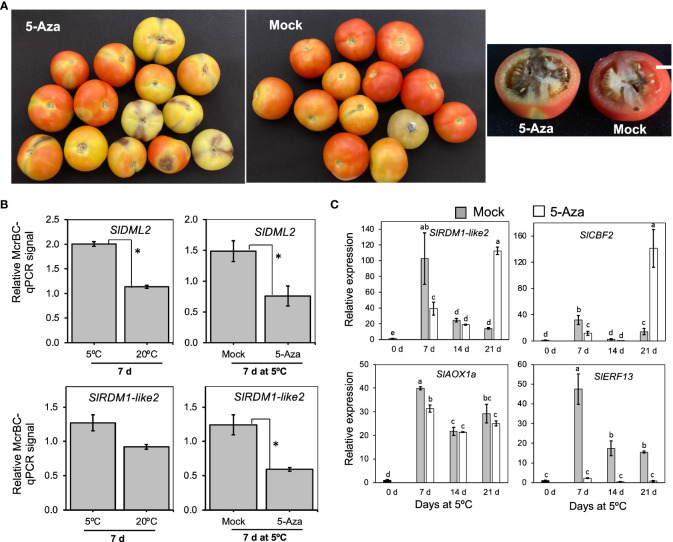
The importance of DNA methylation in cold response and chilling injury development in tomato fruit. **(A)** Images of “Moneymaker” tomato fruits that were treated with the DNA methylation inhibitor, 5-Azacytidine (5-Aza) or mock (distilled water) during storage at 5°C for 21 d followed by 7 d holding at 20°C. White horizontal bars indicate 1cm. **(B)** DNA methylation levels of *SlDML2* and *SlRDM1-like 2*. McrBC-qPCR analysis was carried out in fruits stored at 5°C or 20°C for 7 d, as well as in mock or 5-Aza-treated samples after 7 d at 5°C. McrBC digests methylated DNA, thus higher McrBC-qPCR signals indicate higher Ct values and hence higher methylation levels. Asterisks indicate significant differences (*P* < 0.05) **(C)** RT-qPCR analysis of selected low temperature-upregulated genes in mock and 5-Aza-treated samples during storage at 5°C for the indicated times. Expression values are relative to the value at harvest (0 d) and the housekeeping gene was *SlActin*. Datapoints indicate means (± SE) of three replicate fruit. Different letters indicate significant differences in ANOVA (Tukey’s test, *P* < 0.05).

After examining gene expression patterns, we determined the involvement of DNA methylation in cold response and CI development in “Moneymaker” fruits using 5-azacytidine, an inhibitor of DNA methylation (Huang et al., 2019; Zhu et al., 2020) and McrBC, a restriction enzyme that cuts methylated DNA. Fruits injected with 5-azacytidine developed relatively severe symptoms (dark spots on the skin and darkening of columella) compared to those injected with distilled water (mock) (**Figure 9A**). Furthermore, fruits injected with 5-azacytidine were generally firmer at the end of cold storage than mock fruits (**Supplementary Figure 2**). We then assayed the DNA methylation levels of *SlDML2* and *SlRDM1-like 2* using McrBC-qPCR analysis. Genomic DNA was first extracted from the pericarp of fruits stored at either 5°C or 20°C for 7 d and then digested with McrBC before performing qPCR using both digested and undigested DNA as templates. Results revealed that methylation levels of both *SlDML2* and *SlRDM1-like 2* were higher in samples at 5°C than at 20°C, and treatment with 5-azacytidine significantly lowered the methylation levels at 5°C with respect to mock samples (**Figure 9B**). To further investigate the influence of 5-azacytidine treatment on gene expression, we examined the transcript levels of 4 genes induced by low temperature alone which we previously identified by RNA-Seq analysis. As shown in **Figure 9C**, 5-azacytidine treatment decreased the expression of *SlRDM1-like 2*, *SlCBF2*, and *SlAOX1a* only after 7 d. However, it is intriguing that the inhibitor completely decreased the expression of *SlERF13* throughout the 21 d of storage at 5°C (**Figure 9C**). These results show that DNA methylation has an important role in cold responses and CI development in tomato fruits.

## 4 Discussion

Cold stress responses and the underlying mechanisms have been well deliberated in the vegetative plant organs (Kidokoro et al., 2022), but conclusions from these studies cannot be directly extrapolated to fruits partly because ripening responses cloud our view. In this work, cold stress studies were performed in tomato fruits at the mature green stage to avoid the effects of autocatalytic ethylene (system II ethylene), which is produced at later maturity stages and largely controls fruit ripening. Additionally, treatments with 1-MCP were done regularly during cold storage to inhibit any probable effects of system I ethylene. Ultimately, we show by RNA-Seq analysis that low temperature triggers defined transcriptional adjustments, including those associated with epigenetic modifications, NcRNA metabolism, ribosome biogenesis, proteosome, alternative splicing and a certain set of transcription factors, which are clearly distinct from fruit ripening-related changes.

Tomatoes are typical climacteric fruits and thus ripening-associated are largely regulated by the phytohormone ethylene (Li et al., 2019). Ethylene-controlled ripening responses are completely inhibited by 1-MCP (Watkins, 2006), a synthetic chemical that boasts a competitive edge over ethylene for receptor binding sites (Blankenship and Dole, 2003). In agreement with these previous findings, our results show that peel color changes from green to red, which occurred in non-treated tomato fruits during storage at 20°C (**Figures 1A, C**), were accompanied by a sharp increase in ethylene production rates (**Figure 1D**; **Supplementary Figure 1**). These changes were strongly inhibited in the 1-MCP-treated fruits. Recovery of ethylene sensitivity and fruit ripening following 1-MCP treatment, likely due to ethylene receptor turnover, has been reported previously (Watkins, 2006). The recovery periods vary depending on fruit species but in tomatoes, Opiyo and Ying (2005) demonstrated that it occurs 2 d after treatment with 1-MCP. In the present study, 1-MCP treatments were carried out 2–3 times per week to overcome possible recovery and hence keep the fruits insensitive to ethylene throughout the experimental duration. As a result, fruit ripening was completely inhibited during the first 14 d (**Figure 1A**), and even though ethylene production rates increased afterwards, the fruits remained yellow and failed to attain the red ripe stage throughout the 35-d storage period. The role of ethylene in fruit ripening and the inhibitory effect of 1-MCP are further confirmed by the identification of module VI genes by RNA-Seq analysis. Precisely, module VI genes were up- or down-regulated at 20°C (**Figure 2B**) in the presence of large ethylene amounts (**Figure 1D**), showed insignificant changes at 5°C, and comprised well-known fruit ripening regulators such as *SlRIN*, *SlNOR*, and *SlCNR* (**Figure 3**). 1-MCP treatments also abolished these expression changes at 20°C, confirming that the genes were under ethylene regulation. Interestingly, *SlSGR* (*Solyc12g056480*) failed to respond to ethylene (at 20°C), but its transcript levels increased appreciably at 5°C (**Figure 3A**), indicating that it is regulated by low temperature most likely in an ethylene-independent manner. This finding agrees with our previous findings in lemons (Mitalo et al., 2020), Satsuma mandarins (Mitalo et al., 2022), and kiwifruits (Mitalo et al., 2019a), where we demonstrated that the expression of certain fruit ripening-associated genes is modulated by low temperature independently of ethylene.

Like other cold sensitive crops, tomatoes develop CI symptoms when stored at sub-optimum low temperatures (< 10°C). Common CI symptoms, including impaired or blotchy ripening, surface pitting and decay, often occur during rewarming after cold storage (Biswas et al., 2016). In the present study, both “Micro-Tom” and “Moneymaker” fruits also showed CI symptoms during rewarming at 20°C following storage at 5°C (**Figures 1B, E, F**; **Supplementary Figure 1**), regardless of whether ethylene signaling was inhibited (by 1-MCP treatment) or not. RNA-Seq analysis revealed that cold stress causes a greater transcriptome response than fruit ripening, as the expression of more genes was altered after 14 d at 5°C (8,406) than at 20°C (4,814) (**Figure 2A**). It is however intriguing that 3,244 genes (in module IX) showed insignificant expression changes at 20°C, but they were differentially expressed at 5°C (**Figure 2B**), which implied that they responded to a low temperature stimulus. Furthermore, the observation that repeated 1-MCP treatments failed to inhibit their expression at 5°C, indicated that their regulation by low temperature is independent of ethylene signaling. It is noteworthy that the expression of a considerable number of genes which are associated with oxidative damage, an indirect outcome of cold stress and abiotic stress in general, was also triggered uniquely by low temperature (**Figure 4**). Transcripts of *SlAOX1a* and several *SlPOXs* were also previously shown to accumulate in tomato fruits during cold storage (Cruz-Mendívil et al., 2015; Albornoz et al., 2019). However, the present study is the first time we are demonstrating that several of these redox genes respond uniquely to cold stress. Furthermore, the existence of low temperature-induced and ethylene-independent gene expression has been previously reported in kiwifruits (Mitalo et al., 2019a), “Passe Crassane” pears (Mitalo et al., 2019b), and some citrus fruit species (Mitalo et al., 2020; Mitalo et al., 2022), but to the best of our knowledge, this study is the first report of such a phenomenon in tomato fruits.

We then used GO and KEGG pathway enrichment analysis to identify molecular processes that are uniquely triggered by cold stress in tomato fruits. The results pointed out that ribosome biogenesis, proteasome, spliceosome and regulation of gene expression are relevant processes (**Figures 2C, D**, **5**, **6**). A notable member of the ribosome biogenesis group was *SlZFP622/REIL1* (**Figure 5C**), while *SlSmEb* stood out among the spliceosome genes (**Figure 6C**). In Arabidopsis, *AtREIL2* encodes a cytosolic ribosomal 60S-biogenesis factor which was shown to activate the biosynthesis of specialized ribosomes during cold acclimation (Schmidt et al., 2013; Cheong et al., 2021), whereas AtSmEb was recently shown to control the splicing of pre-mRNAs during chilling stress (Wang et al., 2022). An understanding of why these genes are activated in tomato fruits under cold stress, yet the fruits still develop CI symptoms requires further research. The global transcriptome analysis revealed, moreover, that several genes encoding transcription factors, chaperones, hormone signaling, and small signaling peptides are induced uniquely by cold stress in tomato fruits (**Supplementary Table 11**). Furthermore, qPCR analysis confirmed their low temperature-specific responsiveness (**Figure 7**), as their induction was swiftly and consistently reversed upon rewarming of the fruits. Orthologs of some of these genes, including *SlPIF3*, *SlJAZ2* and *SlERF13*, have been linked with cold tolerance in leaves of other plant species (Jiang et al., 2017; Li et al., 2017; Lv et al., 2020). However, the others have not been reported previously even though several CLE peptides have been linked with various abiotic stresses in plants (Kim et al., 2021). It is also worth mentioning that *SlCBF2* showed a clearly cold-specific expression pattern in “Moneymaker” fruits (**Figure 7B**), but in “Micro-Tom” fruits, it was induced by both ethylene and cold stress (**Figure 7A**). The reason for this discrepancy cannot be expounded in this study, as both cultivars displayed CI symptoms at almost the same degree. However, it could be an indicator of minor cultivar differences in terms of the regulation of cold responsive genes.

Epigenetic regulation has been associated with plant responses to a broad range of stresses, including cold stress (Kim et al., 2015). In this respect, Zhang et al. (2016) demonstrated that cold stress triggers an increase in DNA methylation in red ripe tomatoes, which significantly affects the expression patterns of various ripening-related genes. In the present study, RNA-Seq analysis underscored the relevance of epigenetic modifications in cold-specific responses in tomato fruits. A considerable number of genes involved in *de novo* DNA methylation and chromatin formation/remodeling were up- or down-regulated at 5°C with ethylene signaling having little or no significant effect on their expression (**Figure 8**), while expression changes at 20°C were also minimal. Notable among these genes was *SlRDM1-like 2*, whose loss-of-function mutations in Arabidopsis showed reduced DNA methylation together with impaired accumulation of 24-nt short-interfering RNAs (Gao et al., 2010). Another major change was the exclusive accumulation of transcripts related with dicer-like enzymes, particularly *SlDCL2d*, during storage at 5°C (**Figure 8**). *SlDCL2s* have been widely studied in terms of plant defense against viral pathogens (Wang et al., 2018; Suzuki et al., 2019; Alcaide et al., 2022). Hunter et al. (2021) also demonstrated that transcripts of both *SlDCL2b* and *SlDCL2d* accumulated to a great degree in ripe cherry tomato fruits during cold storage, but our present study is the first to show that this accumulation is unique to cold stress and is independent of ethylene signaling. Treatment of mature green tomato fruit with the DNA methylation inhibitor, 5-azacytidine, confirmed the existence of cold stress-induced methylation dynamics and its influence on gene expression (**Figure 9**). While 5-azacytidine effects are more pronounced in actively dividing cells (Jones and Taylor, 1980), recent reports have demonstrated the effectiveness of the chemical in studying methylation dynamics even in mature fruits having non-dividing cells (Huang et al., 2019; Zhu et al., 2020). Nevertheless, one of the major limitations of using 5-azacytidine is that it induces cytotoxicity. In the present study, we did not detect toxicity symptoms in 5-azacytidine-treated fruits at 20°C for up to 14 d (data not shown), but at 5°C treated fruits appeared to exhibit severe ripening inhibition symptoms than mock fruits. It is therefore possible that a complex interaction between cold stress and 5-azacytidine and this needs to be studied further. All in all, further research is required to understand the role and involvement of the cold-specific *SlRDM1-like 2* as well as *SlDCLs* in *de novo* DNA methylation (as shown in **Figure 9**) and RNA silencing mechanisms during cold responses and CI development in tomato fruits.

A diverse number of genes have been shown to be likely involved in cold tolerance in tomato plants. However, although it was demonstrated that *LeGPA1* and *LeCOR413PM2* are induced by cold stress in tomato leaves (Guo et al., 2020; Zhang et al., 2021), both genes were downregulated with no cold-specific expression in the fruit pericarp samples analyzed in the present study. A possible explanation is that cold stress-induced gene expression can differ due to variations in tissue-specific physiological and developmental responses, as previously reported in tomato (Weiss and Egea-Cortines, 2009), as well as other plant species (Sanchez-Ballesta et al., 2004; Xin et al., 2019). In the present study, most of the cold-specific genes identified in the fruits showed similar expression patterns in the leaves (**Supplementary Figure 3**), suggesting their involvement in cold responses in both tissues.

In conclusion, we have utilized RNA-Seq analysis to pinpoint the transcriptional dynamics triggered by cold stress in tomato fruits, particularly those which are clearly distinct from ethylene signaling (and by extension, fruit ripening)-mediated ones. We have further demonstrated the likely involvement of *de novo* DNA methylation in the regulation of these cold-specific transcriptional changes. Other cold-specific transcriptional adjustments like the spliceosome, ribosome biogenesis, peptide biosynthesis and proteasomal catabolism point towards a strong re-alignment to alter the protein composition of tomato fruit cells in response to cold stress. These changes present potential regulators and players in the regulatory network for cold stress sensing and signaling pathways in fruits. Furthermore, considering that these changes do not occur during normal fruit ripening (at 20°C), the genes identified in the present study will encourage further research on genetic improvement of cold tolerance and CI reduction, without having to worry about undesirable effects on fruit ripening.

## Data availability statement

The data presented in the study are deposited in the DNA Data Bank of Japan (DDBJ) repository, accession numbers DRR461198-DRR461224.

## Author contributions

OM, HE, and YK conceived and designed the study. OM did most of the experiments with close supervision from HE, TA, and SK. LT provided technical assistance especially with growing of plants and data analysis. OM wrote the first draft of the manuscript which was substantially improved by all authors. All authors contributed to the article and approved the submitted version.

## Footnotes

1
https://usegalaxy.org/

2
http://bioinfo.ut.ee/primer3-0.4.0/

3
www.r-project.org
