# Corrigendum: Transcriptomic analysis in tomato fruit reveals divergences in genes involved in cold stress response and fruit ripening

**DOI:** 10.3389/fpls.2024.1421564

**Published:** 2024-04-30

**Authors:** Oscar W. Mitalo, Seung Won Kang, Long T. Tran, Yasutaka Kubo, Tohru Ariizumi, Hiroshi Ezura

**Affiliations:** ^1^ Graduate School of Life and Environmental Sciences, University of Tsukuba, Tsukuba, Japan; ^2^ Tsukuba-Plant Innovation Research Center, University of Tsukuba, Tsukuba, Japan; ^3^ Graduate School of Environmental and Life Science, Okayama University, Okayama, Japan

**Keywords:** cold stress, chilling injury, “Micro-Tom”, “Moneymaker”, RNA-Seq, ripening

In the published article, an author name was incorrectly written as Seung Wong Kang. The correct spelling is Seung Won Kang.

The authors apologize for this error and state that this does not change the scientific conclusions of the article in any way. The original article has been updated.

