# Supplementary material for: Transcriptomic analysis in tomato fruit reveals divergences in genes involved in cold stress response and fruit ripening
Source: Front Plant Sci. 2023 Jul 28;14:1227349. doi: 10.3389/fpls.2023.1227349 (PMC10416649; doi:10.3389/fpls.2023.1227349)
Supplement: Supplementary file 1 [file DataSheet_1.zip › Supplementary material_1/Supplementary Figure 3.pptx]

## Slide 1
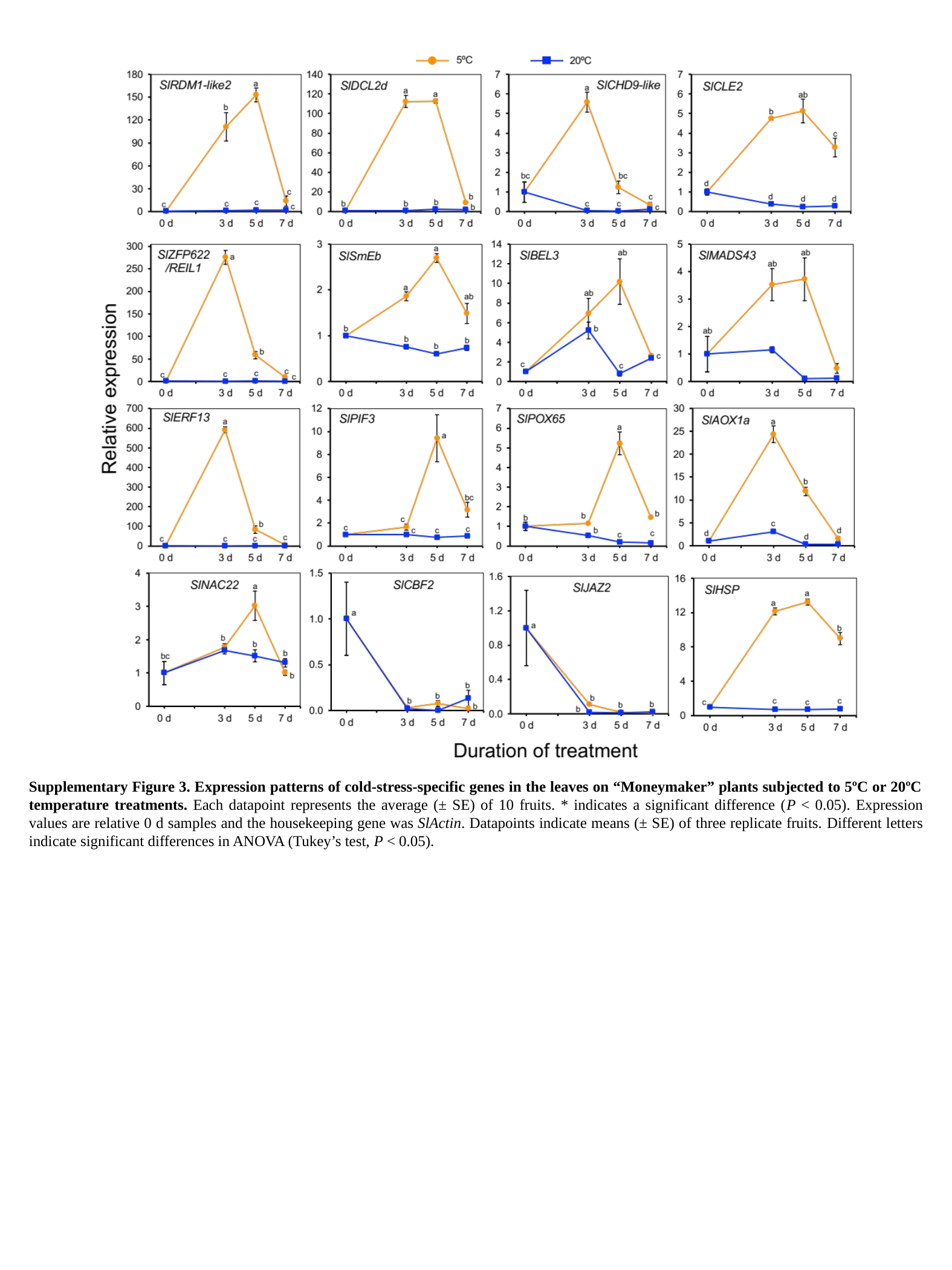

Supplementary Figure 3. Expression patterns of cold-stress-specific genes in the leaves on “Moneymaker” plants subjected to 5ºC or 20ºC temperature treatments. Each datapoint represents the average (± SE) of 10 fruits. * indicates a significant difference (P < 0.05). Expression values are relative 0 d samples and the housekeeping gene was SlActin. Datapoints indicate means (± SE) of three replicate fruits. Different letters indicate significant differences in ANOVA (Tukey’s test, P < 0.05).
