# Supplementary material for: Transcriptomic analysis in tomato fruit reveals divergences in genes involved in cold stress response and fruit ripening
Source: Front Plant Sci. 2023 Jul 28;14:1227349. doi: 10.3389/fpls.2023.1227349 (PMC10416649; doi:10.3389/fpls.2023.1227349)
Supplement: Supplementary file 1 [file DataSheet_1.zip › Supplementary material_1/Supplementary Figure 2.pptx]

## Slide 1
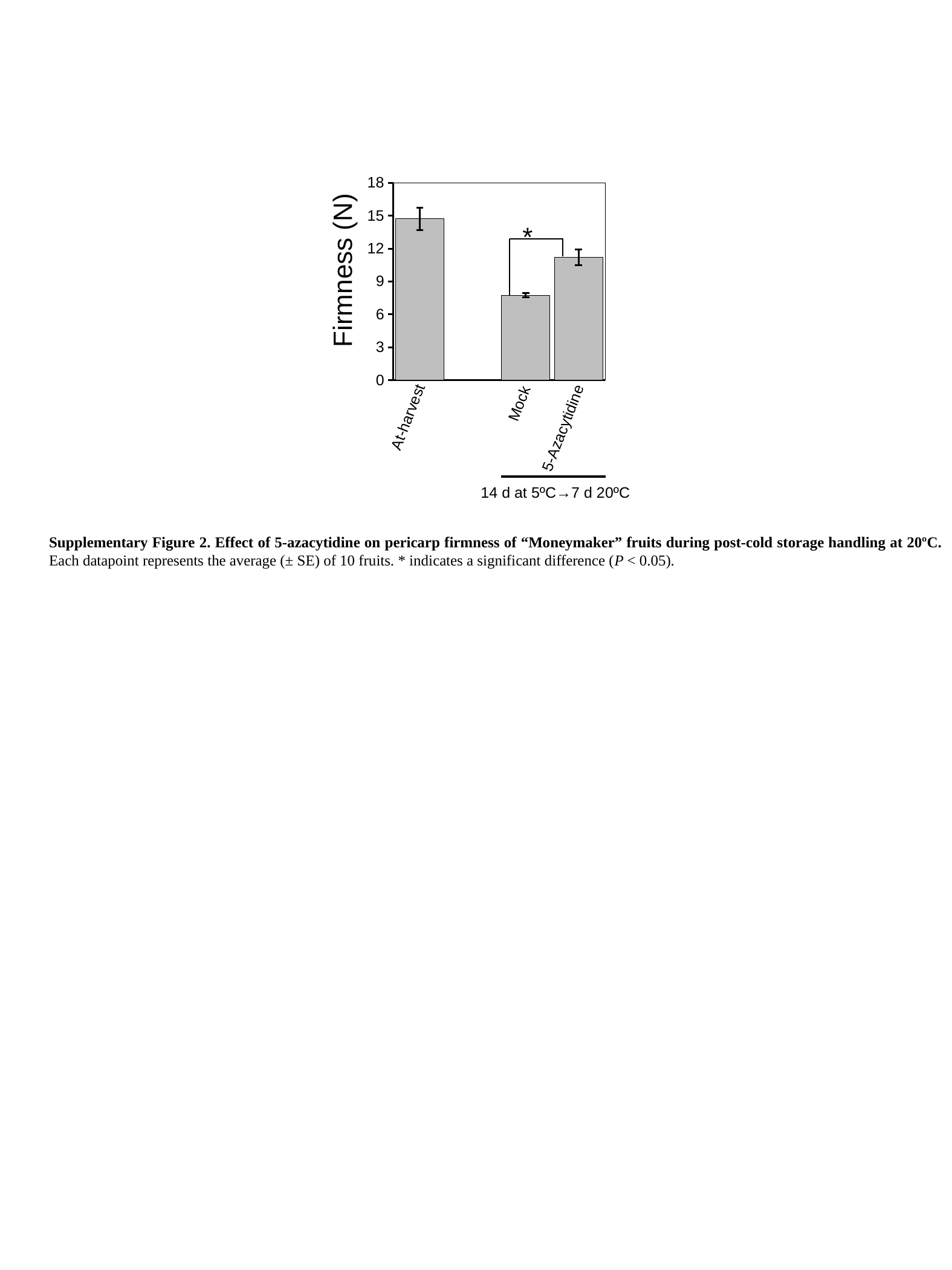

### Chart
| Category | |
|---|---|
| At-harvest | 14.72166666666667 |
| | None |
| Mock | 7.745 |
| 5-AZA | 11.213333333333333 |Mock
At-harvest
5-Azacytidine
14 d at 5ºC→7 d 20ºC
*
Firmness (N)
Supplementary Figure 2. Effect of 5-azacytidine on pericarp firmness of “Moneymaker” fruits during post-cold storage handling at 20ºC. Each datapoint represents the average (± SE) of 10 fruits. * indicates a significant difference (P < 0.05).
