# Supplementary material for: Transcriptomic analysis in tomato fruit reveals divergences in genes involved in cold stress response and fruit ripening
Source: Front Plant Sci. 2023 Jul 28;14:1227349. doi: 10.3389/fpls.2023.1227349 (PMC10416649; doi:10.3389/fpls.2023.1227349)
Supplement: Supplementary file 1 [file DataSheet_1.zip › Supplementary material_1/Supplementary Figure 1.pptx]

## Slide 1
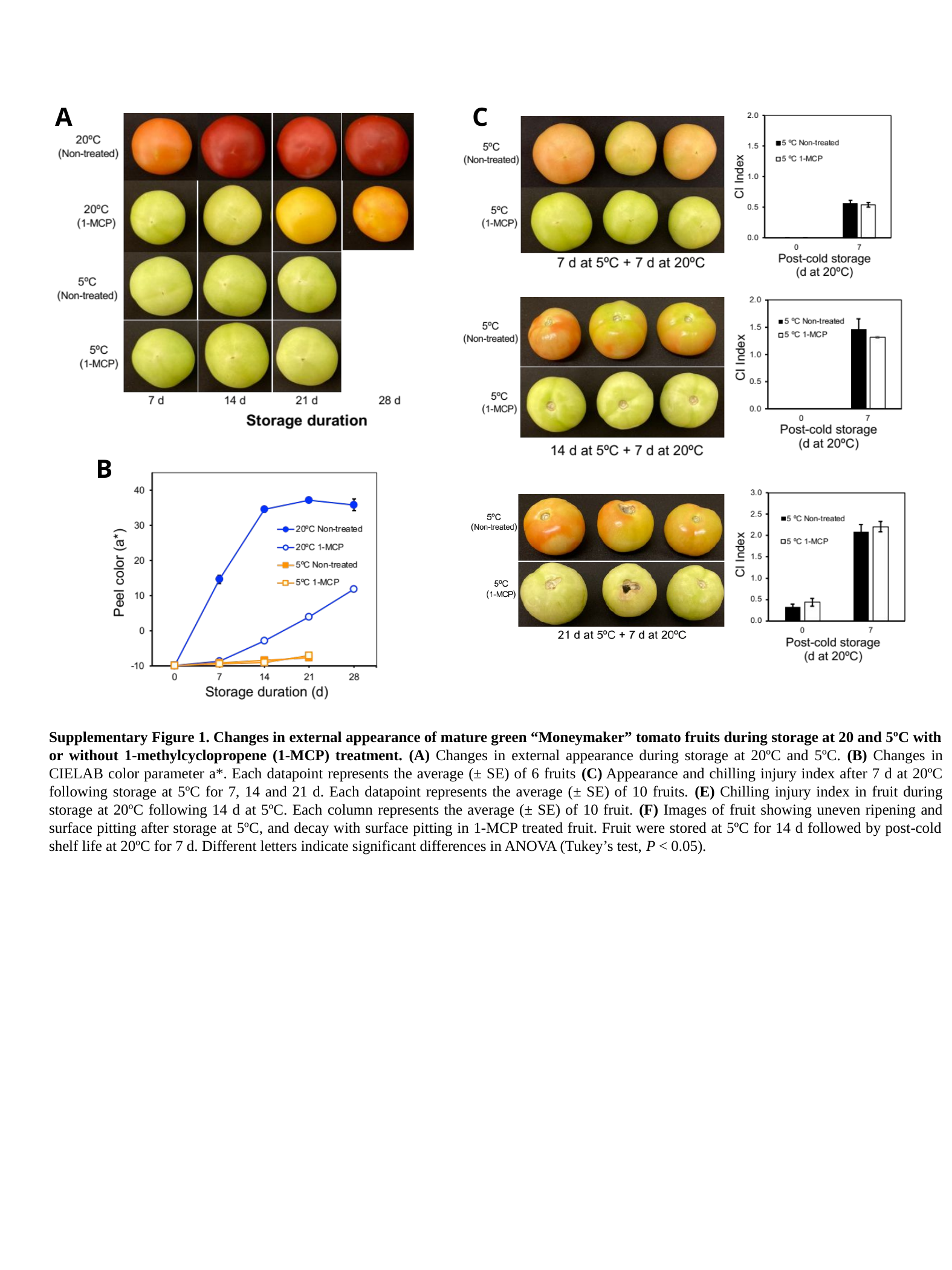

A
C
B
Supplementary Figure 1. Changes in external appearance of mature green “Moneymaker” tomato fruits during storage at 20 and 5ºC with or without 1-methylcyclopropene (1-MCP) treatment. (A) Changes in external appearance during storage at 20ºC and 5ºC. (B) Changes in CIELAB color parameter a*. Each datapoint represents the average (± SE) of 6 fruits (C) Appearance and chilling injury index after 7 d at 20ºC following storage at 5ºC for 7, 14 and 21 d. Each datapoint represents the average (± SE) of 10 fruits. (E) Chilling injury index in fruit during storage at 20ºC following 14 d at 5ºC. Each column represents the average (± SE) of 10 fruit. (F) Images of fruit showing uneven ripening and surface pitting after storage at 5ºC, and decay with surface pitting in 1-MCP treated fruit. Fruit were stored at 5ºC for 14 d followed by post-cold shelf life at 20ºC for 7 d. Different letters indicate significant differences in ANOVA (Tukey’s test, P < 0.05).
